# Proteomic Analysis Reveals Age-related Changes in Tendon Matrix Composition, with Age- and Injury-specific Matrix Fragmentation*[Fn FN2]

**DOI:** 10.1074/jbc.M114.566554

**Published:** 2014-07-30

**Authors:** Mandy J. Peffers, Chavaunne T. Thorpe, John A. Collins, Robin Eong, Timothy K. J. Wei, Hazel R. C. Screen, Peter D. Clegg

**Affiliations:** From the ‡ Department of Musculoskeletal Biology, Institute of Ageing and Chronic Disease, University of Liverpool, Leahurst Campus, Neston CH64 7TE,; § Institute of Bioengineering, Queen Mary University of London, Mile End Road, London E1 4NS, and; ¶ School of Life Sciences, Ngee Ann Polytechnic, Singapore 599489

**Keywords:** Aging, Collagen, Mass Spectrometry (MS), Protein Degradation, Proteoglycan, Disease, Equine, Fibromodulin, Neopeptide

## Abstract

Energy storing tendons, such as the human Achilles and equine superficial digital flexor tendon (SDFT), are highly prone to injury, the incidence of which increases with aging. The cellular and molecular mechanisms that result in increased injury in aged tendons are not well established but are thought to result in altered matrix turnover. However, little attempt has been made to fully characterize the tendon proteome nor determine how the abundance of specific tendon proteins changes with aging and/or injury. The aim of this study was, therefore, to assess the protein profile of normal SDFTs from young and old horses using label-free relative quantification to identify differentially abundant proteins and peptide fragments between age groups. The protein profile of injured SDFTs from young and old horses was also assessed. The results demonstrate distinct proteomic profiles in young and old tendon, with alterations in the levels of proteins involved in matrix organization and regulation of cell tension. Furthermore, we identified several new peptide fragments (neopeptides) present in aged tendons, suggesting that there are age-specific cleavage patterns within the SDFT. Proteomic profile also differed between young and old injured tendon, with a greater number of neopeptides identified in young injured tendon. This study has increased the knowledge of molecular events associated with tendon aging and injury, suggesting that maintenance and repair of tendon tissue may be reduced in aged individuals and may help to explain why the risk of injury increases with aging.

## Teaser

**Background:** Alterations in tendon matrix composition with aging and injury are poorly understood.

**Results:** Aging and injury resulted in distinct protein profiles, with age-specific peptide fragmentation in injury.

**Conclusion:** Identification of protein cleavages associated with aging and injury suggest impaired maintenance and repair in aged tendon.

**Significance:** Novel peptide fragments identified are potential biomarkers of tendon injury and age-related degeneration.

## Introduction

Current descriptions of tendon extracellular matrix (ECM)^4^ in the literature list the main components as collagen type I, proteoglycans (predominantly small leucine-rich proteoglycans (SLRPs)) (1), minor collagens (types III, V, VI, XII) (2), elastic fibers (3), and glycoproteins (4). The most abundant protein, collagen type I, aligns with the tendon long axis and aggregates in a series of hierarchical levels to form fibrils, fibers, fascicles, and finally the whole tendon (5). At the larger hierarchical levels the collagen is interspersed with a proteoglycan-rich matrix. This multilevel fiber composite organization results in a tendon with high uniaxial strength that is able to resist the large tensional forces experienced *in vivo*.

It is well established that tendon functional integrity decreases with aging, predisposing aged tendons to degeneration and injury (6, 7). Additional risk factors for tendon injury include high levels of repetitive loading (8), genetic factors (9, 10), and chronic inflammation (11). However, the cellular and molecular mechanisms underpinning this increased risk in tendons are not well understood. Several studies have reported alterations in matrix content as a function of aging, including increased type III collagen (12), changes in cross-link profile as a result of glycation (13, 14), and accumulation of partially degraded collagen within the matrix (14). Age-related alterations to the non-collagenous matrix have also been identified, with decreased glycosaminoglycan and cartilage oligomeric matrix protein (COMP) levels (15, 16). Several of these studies indicate altered matrix turnover with aging. In cartilage, ECM fragmentation patterns have demonstrated novel potential substrates and cleavage sites for specific enzymes (17). Although a recent study has identified stage-specific peptide fragments in tendon disease (18), there is also a need to identify age-specific cleavage sites in tendon as this will enable the understanding and distinction of the ECM degradative mechanisms associated with aging and disease.

It is important to further characterize the tendon ECM and identify aging changes in both health and disease, as it is likely that alterations to minor matrix components may have a profound influence on tendon function. However, some minor components of the tendon matrix may not yet have been identified. Although proteomic analysis has been used to identify many novel proteins in other connective tissues such as cartilage (19, 20), a review of the current literature shows few studies that have undertaken a proteomic analysis of tendon. Considering the studies that have addressed tendon proteomics, some have assessed the proteins produced by tendon fibroblasts *in vitro* (21, 22), whereas others have investigated alterations in protein profile as a result of artificially induced injury (23, 24). Smith *et al.* (25) investigated changes in pericellular proteins during development, and Dakin *et al.* (18) studied normal and diseased tendons from horses with a wide age range but do not report any data regarding age-related alterations in protein content. To the authors' knowledge, no studies have assessed age- and injury-associated changes in the tendon extracellular matrix protein profile.

In the current study we used equine tendon tissue to study the effect of aging and injury on tendon matrix composition. The horse is an accepted and relevant model in which to study musculoskeletal aging and injury, as it is a relatively long-lived species in which age-related musculoskeletal diseases, such as tendon injury, show a very similar epidemiology, etiology, and pathology to that seen in human age-related musculoskeletal diseases (14, 26–30). In both species the most commonly injured tendons are those that store and return energy during locomotion. In the human it is the Achilles tendon that is the major energy store and the most prone to injury (31), whereas in the horse, the predominant energy store is the superficial digital flexor tendon (SDFT) (32). We, therefore, assessed the protein profile of normal and injured SDFTs from young and old horses using label-free relative quantification to identify differentially abundant proteins between age groups. Furthermore we investigated age-specific cleavage patterns in the ECM by assessing fragmentation patterns of specific matrix molecules to identify neopeptides in injured and aged tendon. One way to provide new insights into the development and treatment of tendon disease is to obtain an understanding of how tendon undergoes the physiological remodeling that is evident in aging. We hypothesized that we would identify age-related alterations in ECM proteins and neopeptides within the tendon matrix, with greater matrix fragmentation evident in injured tendon.

## EXPERIMENTAL PROCEDURES

All chemicals were supplied by Sigma unless otherwise stated.

### 

#### 

##### Tendon Sampling and Procurement

Forelimbs, distal to the carpus, were collected from half to full thoroughbred horses (young, 3.3 ± 0.6 years; old, 19.0 ± 1.7 years, both *n* = 3), euthanized at a commercial equine abattoir. Only tendons that had no evidence of previous tendon injury at post-mortem examination were included in the study. The SDFT was dissected free from the limbs from the level of the carpus to the metacarpophalangeal joint. Fascicles (length of 25 mm, diameter of 0.2–0.4 mm, weight of ∼0.3 g) were dissected in duplicate from the mid-metacarpal region of the tendon as described previously (33). The fascicles were snap-frozen in liquid nitrogen and stored at −80 °C until further analysis.

##### Protein Extraction and Sample Preparation

Each thawed tendon sample (fascicle) was transferred into an Eppendorf tube containing 200 μl of 100 mm Tris acetate, protease inhibitors (Complete Protease Inhibitors, EDTA-free, Roche Applied Science), and 0.1 unit of chondroitinase ABC, pH 8.0, and deglycosylated for 6 h at 37 °C. The supernatant was removed after centrifugation at 13,000 × *g* for 5 min. 0.5 ml of guanidine extraction buffer (4 m guanidine hydrochloride (GdnHCl), 65 mm dithiothreitol (DTT), and 50 mm sodium acetate, pH 5.8) was added, and extraction was performed with end-over-end mixing for 48 h at 4 °C. 25 mm DTT was added 2 h before the addition of 80 mm iodoacetamide, the latter for the last 2 h in the dark. The soluble fraction was removed after centrifugation for 15 min at 13,000 × *g* at 4 °C. The final insoluble fraction was incubated in 0.5 ml of 100 mm acetic acid containing 100 μg/ml pepsin overnight at 4 °C with end-over-end mixing to release collagenous polypeptides. The supernatant was removed after centrifugation at 13,000 × *g* for 15 min at 4^°^C. This was lyophilized, resuspended in water, re-lyophilized, and stored at −80 °C. Protein concentrations of aliquots of soluble fraction were estimated by the Bradford assay using Coomassie Plus^TM^ protein assay reagent (Thermo Scientific, Rockford, IL) read at 660 nm after acetone precipitation.

##### One-dimensional Sodium Dodecyl Sulfate Polyacrylamide Gel Electrophoresis (SDS-PAGE) and In-gel Trypsin Digestion

Tendon GdnHCl soluble extracts were analyzed by one-dimensional SDS-PAGE to assess gross quantitative/qualitative differences in protein profiles between young and old tendon. Samples were loaded according to equal volumes after acetone precipitation and resolubilization in buffer containing 8 m urea, 2% (w/v) CHAPS and 0.0002% (v/v) bromphenol blue plus 0.2% (v/w) DTT.

Aliquots were heated in Laemmli buffer containing 50 mm DTT for 5 min at 95 °C and resolved through 4–12% acrylamide Bis-Tris NuPAGE gels (Invitrogen), and proteins were visualized using a silver staining kit (Thermo Scientific) according to the manufacturer's instructions. In-gel tryptic digestion of dominant bands was undertaken as previously described (34).

To detect pepsin-released collagenous polypeptides, the lyophilized samples were reconstituted in 0.1 m acetic acid containing pepsin at 100 μg/ml and shaken overnight at 4 °C. After centrifugation at 13,000 × *g* for 15 min, the supernatant was removed, lyophilized, and resuspended in water before re-lyophilizing and heating in 20 μl of Laemmli buffer containing 50 mm DTT for 5 min at 95 °C. The material was resolved using 3–8% acrylamide Tris acetate gels (Invitrogen) and silver-stained.

##### Protein Identification of Bands Using LC-MS/MS

Peptides were analyzed using a Bruker Amazon ion trap mass spectrometer coupled to a Waters nanoACQUITY UltraPerformance liquid chromatography system. The samples were injected onto a reverse phase column (Acquity ethylene bridged hybrid C18, 75 μm × 150 mm, 1.7 μm) and eluted over a 1-h gradient. The mass spectrometer was set up in positive ion mode and calibrated with Bruker calibration mix. Spectra were acquired between 300 and 1800 *m*/*z* with an ion charge count target of 200,000. Up to five precursor ions above a threshold of 10,000 were selected for MSMS fragmentation per MS scan. Each precursor was fragmented twice, and then the mass was excluded for 1 min. Singly charged ions were excluded. Data were searched against the *Equus caballus* database; Ensembl database for horse (*E. caballus*; EquCab2.56.pep) using an in-house Mascot server (Matrix Science, London, UK). Parameters were set to accept one miscleavage, a fixed modification of carbamidomethly cysteine, and a variable oxidation of methionine. The peptide mass tolerance for this instrument was set at 0.4 Da.

##### Protein In-solution Trypsin Digestion and Mass Spectrometry Using Linear Ion-trap Orbitrap Mass Spectrometer (LTQ-Orbitrap Velos)

Proteomic analyses were performed to identify cellular and matrix proteins present within normal tendon tissue, the relative levels of these proteins, and also to identify neopeptides of specific ECM proteins. GdnHCl-extracted proteins were washed with 100 mm ammonium bicarbonate to give a final concentration of 0.5 m GdnHCl. Tryptic digestion was undertaken as previously described (20) but with the addition of a top-up of a further 2 μg after 3 h. LC-MS/MS analysis was performed using nanoAcquityTM ultraperformance LC (Waters, Manchester, UK) on-line to an LTQ-Orbitrap Velos mass spectrometer (Thermo-Fisher Scientific, Hemel Hempstead) as previously described (20) via an electrospray ionization ion source containing a 10-μm coated Pico-tip emitter (Presearch LTD, Basingstoke, UK). Aliquots of tryptic peptides equivalent to 300 ng of tendon fascicle protein were loaded onto a 180-μm × 20-mm C18 trap column (Waters) at 5 μl/min in 99% solvent A (water plus 0.1% formic acid) and 1% solvent B (acetonitrile plus 1% formic acid) for 5 min and subsequently back-flushed onto a C18 pre-equilibrated analytical column (75-μm × 15-mm Waters) using a flow rate of 0.3 μl/min. Xcalibur 2.0 software (Thermo-Electron, Hemel Hempstead, UK) was used to operate the LTQ-Orbitrap Velos mass spectrometer in data-dependant acquisition mode. The survey scan was acquired in the Orbitrap with a resolving power set to 30,000 (at 400 *m*/*z*). MS/MS spectra were concurrently acquired on the 20 most intense ions from the high resolution survey scan in the LTQ mass spectrometer. Charge state filtering >1 was used where unassigned precursor ions were not selected for fragmentation. Fragmentation parameters in the LTQ mass spectrometer were: normalized collision energy, 30; activation, 0.250; activation time, 10 ms; minimum signal threshold, 500 counts with isolation width 2 *m/z*.

##### Label-free Peptide Quantification

For label-free quantification of the tendon fascicles the Thermo raw files of the acquired spectra from in-solution tryptic digests of normal young (*n* = 3) and old (*n* = 3) equine tendon fascicles were analyzed by the Progenesis^TM^ LC-MS software (Version 4, Nonlinear Dynamics) for label-free quantification as previously described (20). Briefly, after the selection of a reference sample, the retention times of the other samples were aligned. Feature picking used the top three spectra for each feature. These were exported from Progenesis^TM^-LC-MS and utilized for peptide identification with a locally implemented Mascot server (Version 2.3.01) in the *E. caballus* database. Search parameters used were: 10 ppm peptide mass tolerance and 0.6-Da fragment mass tolerance; one missed cleavage allowed; fixed modification, carbamidomethylation; variable modifications, methionine oxidation, proline oxidation, and lysine oxidation. To maximize the number of quantifiable proteins but simultaneously use an acceptable false discovery rate (FDR), the peptide matches above an identity threshold were adjusted to give an FDR of 1% before the protein identifications being re-imported into Progenesis^TM^.

Mascot determined peptides with ion scores of 20 and above, and only proteins with at least one unique peptide ranked as the top candidate were considered and re-imported into Progenesis^TM^ software. For quantification, only unique peptides were included. Statistical analysis was performed on all detected features using transformed normalized abundances for one-way analysis of variance (ANOVA). All peptides (with Mascot score >23 and *p* < 0.05) of an identified protein were included, and the protein *p* value (one-way analysis of variance) was then performed on the sum of the normalized abundances for all runs. Adjusted analysis of variance values of *p* < 0.05 and additionally regulation of >2-fold or <0.5-fold were regarded as significant.

##### Neopeptide Identification

For neopeptide determination, mass spectrometry data from the in-solution tryptic digests of normal young (*n* = 3) and old (*n* = 3) equine tendon fascicles were analyzed. Neopeptides were identified by searches against the Unihorse database using Mascot. Search parameters used were: enzyme, none; peptide mass tolerances 10 ppm; fragment mass tolerance of 0.6 Da, 1+, 2+, and 3+ ions; missed cleavages 1; instrument type electrospray ionization-TRAP. Modifications included were; fixed, carbamidomethyl cysteine and variable, oxidation of methionine, proline, and lysine. The probability that a match was correct (*p* < 0.05) was determined using the Mascot-derived ion score, where *p* was the probability that the observed match was a random event. As the cost of mass spectrometry analyses of a large number of samples was prohibitive and to have confidence in our analysis, we only included neopeptides in the results if they were identified by Mascot more than once per donor and in ≥2 donors. Patterns of fragmentation were determined for aggrecan, biglycan, decorin, fibromodulin, COMP, lumican, and collagens.

##### Gene Ontology, Pathway Enrichment Analysis, and Protein Network Analysis

The gene symbols for each identified protein in normal tendon were searched in the Ensembl database for horse and converted to the gene symbol of the corresponding human orthologue. The resulting gene list was used for gene ontology (GO) using the Database for Annotation, Visualization, and Integrated Discovery (DAVID) Version 6.7. In addition, the list was used for protein network analysis with the Search Tool for Retrieval of Interacting Genes/Proteins (STRING) tool Version 9.1 (35). The protein interaction maps were created by allowing for experimental evidence in addition to the predicted functional links: co-occurrence, co-expression, databases, and text-mining.

##### Western Blotting Validation of Fibromodulin Abundance

To validate the decreased fibromodulin abundance with aging in normal tendon, soluble proteins were extracted from separate donors (3 young (4 years old) and 3 old (>20 years old)) using methods described previously (20). Briefly, 20 μg of soluble protein extracts were electrophoresed and separated on 4–12% SDS-PAGE gels (Nu-Page, Invitrogen). Nitrocellulose membranes were probed with primary antibodies against the following: mouse polyclonal to fibromodulin (1:2000 dilution, #67596 Abcam) and α-tubulin (1:1000 dilution) (#4074, Abcam, Cambridge, UK) as the loading control. Membranes were washed and incubated in a secondary horseradish peroxidase-conjugated antibody (1:2000 dilution). Blots were imaged using VisionWorksLS image acquisition software package, and band densities were analyzed using ImageJ 1.42. Results were normalized to the loading control.

##### Real-time Polymerase Chain Reaction (RT-PCR) of Keratin Expression

Samples of normal SDFT RNA from an independent cohort (young, 5.7 ± 1.3 years; old, 23.3 ± 3.1 years (both *n* = 7)) were used to assess age-related alterations in keratin gene expression in normal tendon using previously described methods (36). Exon-spanning primer sequences were designed and validated by PrimerDesign Ltd (Southampton, UK) except for the normalization gene glyceraldehyde-3-phosphate dehydrogenase (GAPDH) (37). The primer pairs were for keratin type 2, cytoskeletal 75;KRT75 (forward reverse), and keratin type 2, cytoskeletal 5;KRT5 (forward reverse).

##### Injured Tendon Study

Injured SDFTs were collected from young (6.3 ± 2.1 years, *n* = 3) and old horses (19.5 ± 3.5 years, *n* = 2) euthanized at a commercial equine abattoir. In all tendons injury was localized to the core of the mid-metacarpal region. All injuries were macroscopically graded as mild-to-moderate in severity, with the appearance of a hemorrhagic lesion (38) but without loss of fascicular pattern (39). Fascicles were dissected from the lesion, proteins were extracted, and one-dimensional SDS-PAGE was performed as described for normal tendon. Samples were trypsin-digested and processed for LCMS-MS as described above. Peptides were quantified using Progenesis software, and neopeptides were identified. Protein networks were identified as described above. Due to normal and diseased samples being run at different times, it was not possible to directly compare normal and diseased groups.

##### Statistical Analysis

Statistically significant differences in the number of proteins and gene expression were identified after log_10_ transformation to ensure normal distribution using Student's *t* test. Statistical analyses were undertaken using S-Plus and Excel software.

## RESULTS

### 

#### 

##### SDS-PAGE Comparative Analysis of Protein Extracts

**FIGURE 1.**
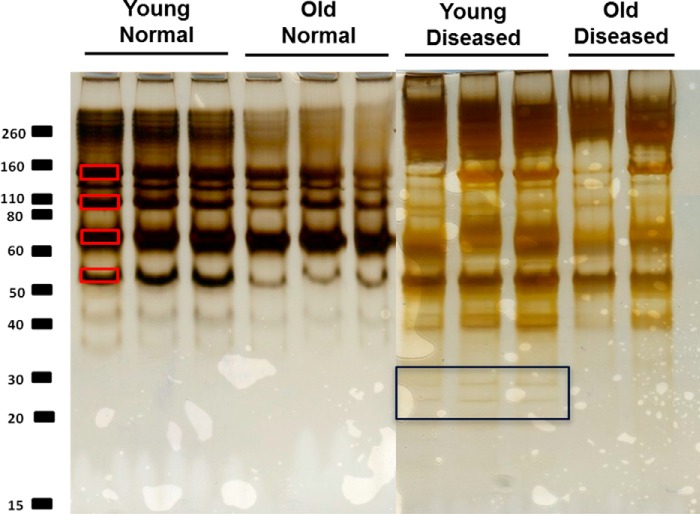
**Silver-stained one-dimensional-SDS-PAGE of the guanidine-soluble protein extract of normal young (*n* = 3) and old (*n* = 3) tendon and diseased young (*n* = 3) and old (*n* = 2).** Equal protein loading by volume (20 μl per well) allowed a qualitative comparison of soluble tendon protein extracts. The most abundant protein bands are marked with *red squares* (1–4) were excised from the gel and trypsin-digested, and the protein content of each single band was analyzed from peptides identified using LC-MS/MS. The *black square* highlights the additional bands evident in diseased young tendon only.

**FIGURE 2.**
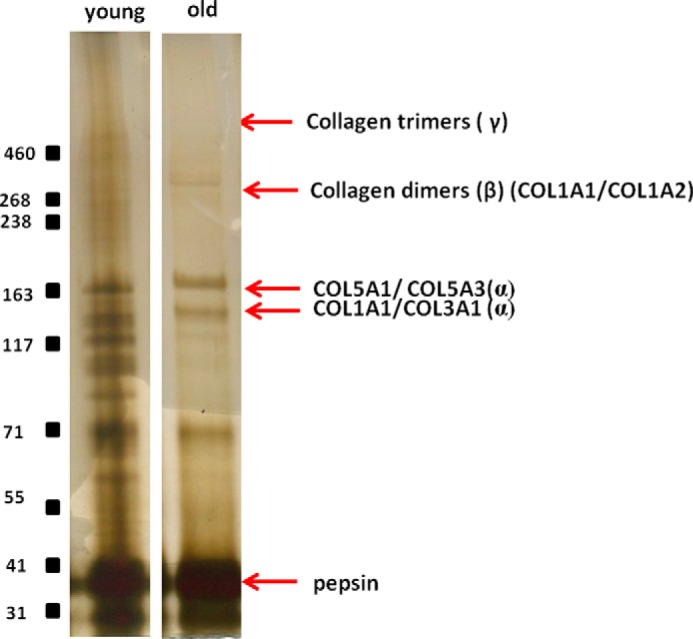
**The entire guanidine-insoluble, pepsin-released material for each sample was resolved by Tris acetate, 3–8% NuPAGE.** The figure shows representative gels for young and old donors. Polypeptides corresponding to the pepsin-released domains of collagen I(αI) (*COL1A1*), collagen I(α2) (*COL1A2*), collagen V(α1) (*COL5A1*), collagen V(α3) (*COL5A3*), collagen III(α1) (*COL3A1*) and cross-linked collagen dimers and trimers are indicated.

One-dimensional-SDS-PAGE of the GdnHCl soluble protein extracts demonstrated differences in the intensity of the staining between samples from normal young and old horses (Fig. 1). The decreased staining in old samples suggests that the extractability of proteins was reduced in older tendon. However, the soluble protein concentrations, corrected to wet weight of tendon fascicle, did not decrease with increasing age (53 ± 1.2 μg/mg for young and 51 ± 0.6 μg/mg for old). The major proteins identified in each band using LC-MS/MS are indicated in Table 1. In addition, we undertook pepsin digestion of the insoluble extract remaining after GdnHCl extraction to analyze non-soluble collagenous polypeptides (19). A number of additional bands were evident in the pepsin digest of young tendon (Fig. 2).

**TABLE 1:** **Proteins identified following in-gel trypsin digestion of bands 1–4 of the soluble guanidine extract using LC-MS/MS** The table lists the most prominent proteins identified following significant peptide matches based on Mascot probability based scoring (*p* < 0.05).

Gel slice	Protein accession	Protein description	Protein score	Protein mass	Protein matches	Protein matches significant	EmPai*^a^*
				*Da*			
1	F6UW03	Collagen α-1(VI) chain	793	110,045	55	38	0.65
	F7CGV8	Collagen α-2(VI) chain	680	110,009	53	34	0.75
	F6QAT0	Collagen α-3(VI) chain	177	343,977	21	10	0.07
	F6YR34	Thrombospondin-1	111	133,474	5	5	0.08
	F7AQV3	Uncharacterized KIAA1211 protein	49	139,560	20	1	0.02
	F6U4X2	α-Fetoprotein	41	70,131	5	2	0.02
2	F6U3D3	Cartilage oligomeric matrix protein	539	84,675	59	32	0.77
	F7E0P3	Thrombospondin-4	69	98,508	11	4	0.08
	F7CGV8	Collagen α-2(VI) chain	45	110,009	1	1	0.03
	F6QAT0	Collagen α-1(VI) chain	35	110,045	2	1	0.03
3	F6RZ46	Prolargin	99	43,846	11	3	0.24
	F6U4X2	α-Fetoprotein	45	70,131	4	3	0.04
	A2Q126	Fibromodulin	35	43,407	7	2	0.16
4	O46542	Decorin	152	40,256	27	13	0.88

*^a^* Exponentially modified protein abundance index (emPAI) approximates label-free relative quantification of proteins in a mixture based on protein coverage by peptide matches.

##### Protein Identification and Ontology

A total of 252 proteins were identified in combined samples from normal tendon; 230 with a significant Mascot score of >23. Supplemental Table 1 provides detailed information on the identification of peptides mapped to each protein and corresponding Mascot scores. When the mgf files for each trypsin-digested sample were analyzed on an individual basis, there was significant variability in the number of proteins identified in normal young and old tendon by Mascot; mean ± S.E.; young 94.5 ± 8.1 and old 58.6 ± 5.1 proteins; *p* = 0.007.

**FIGURE 3.**
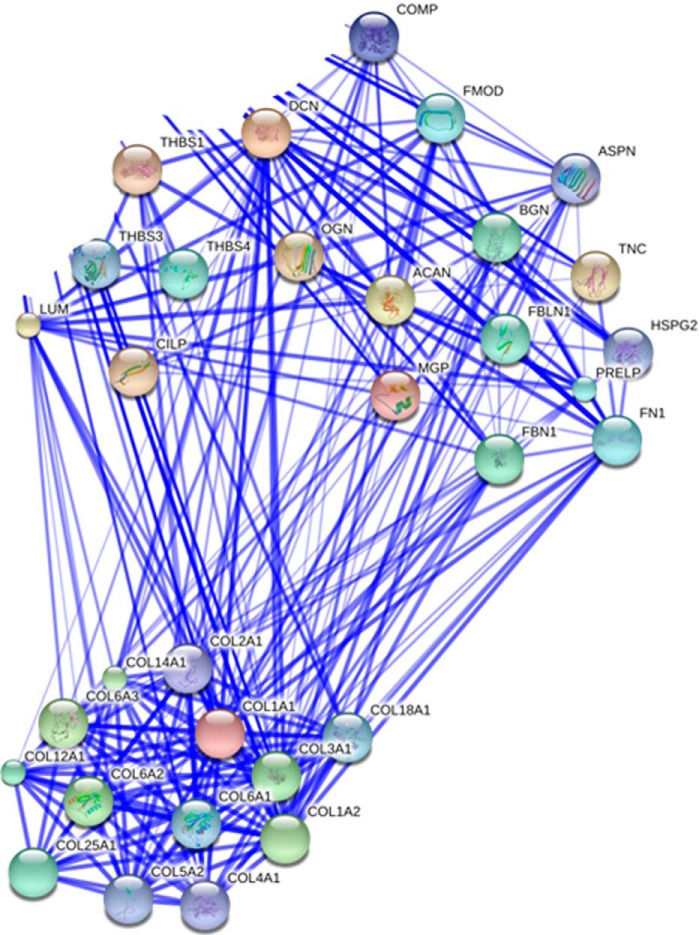
**Protein-protein interaction map of soluble GdnHCl-extracted proteins in normal tendon.** Proteins were input from the total dataset. Unconnected nodes and proteins not relating to the two clusters of matrix organizational proteins and collagens were removed to enable clarity of the interactome. The total cluster was built with STRING allowing for experimentally verified and predicted protein-protein interactions at high confidence levels (0.700). Two highly connected clusters were evident.

For normal tendon the total dataset with a significant Mascot score was transformed to a non-redundant gene identifier list of the respective human homologues and then subjected to gene ontology using DAVID and analysis for protein networks by STRING. A total of 189 equivocal human gene names were used for bioinformatics analysis. These were classified according to their GO annotation as intermediate filament 15%, extracellular matrix 19%, and keratin 12%. DAVID and STRING identified two significant Kegg pathways from the data set; ECM receptor interaction and focal adhesion (Bonferroni adjusted *p* values of 2.44e^−13^ and 2.61e^−10^, respectively) (supplemental Table 2). STRING analyses resulted in a loose network of proteins containing two highly connected clusters around collagen fibril organization and ECM organization (Fig. 3).

##### Identification of ECM Fragmentation Patterns

A catalogue of age-related neopeptides was identified for COMP, decorin, lumican, collagen α-2(I), collagen α-2(VI), collagen α-3(VI), and collagen α-1(XII). These included those identified either in old normal tendon only, young only, and young and old tendon (Table 2).

**TABLE 2:** **Number of neopeptides identified in normal old tendon only, young tendon only, and young and old tendon** Neopeptides were identified by Mascot with a significant score more than once per donor and in ≥2 donors. Numbers in parentheses indicate number of peptides also identified in diseased tendon. For full details of neopeptide sequences, see supplementary information (supplemental Table 3).

Condition	Protein	Number of neopeptides identified
**Normal tendon**		
Old tendon only	Decorin	1 (1)
	COMP	4 (2)
	Col1A2	1
	Col6A2	1
	Col6A3	2
	Col12A1	2
Young tendon only	Decorin	1 (1)
	COMP	6 (2)
	Lumican	1
	Col6A3	7 (1)
	Col12A1	6 (1)
Young and old tendon	Decorin	2 (2)
	COMP	2 (1)
	Lumican	1
	Col6A2	1
	Col6A3	1

**Diseased tendon**		
Old tendon only	Biglycan	4
	Lumican	2
	Col6A1	1
	Col6A3	2
Young tendon only	Aggrecan	4
	Biglycan	5
	COMP	18 (1)
	Decorin	15 (2)
	Fibromodulin	2
	Lumican	4
	Col6A1	3
	Col6A2	6
	Col6A3	12 (1)
	Col12A1	1 (1)
	Col14A1	1
Young and old tendon	Biglycan	2
	COMP	5 (2)
	Decorin	6 (2)
	Lumican	2
	Col6A1	2
	Col6A3	4

##### Label-free Relative Quantification

To compare relative protein levels between normal young and old tendon, samples were processed for LC-MS/MS, and quantitative analysis was undertaken with Progenesis^TM^. Principal component analysis of all the peptides identified revealed that the peptides clustered according to the age of donor, with a principal component of 83%. Levels of 34 proteins differed between young and old tendon (25 with ≥2 peptides). 15 proteins were higher in young tendon (10 with ≥2 peptides), and 19 proteins were higher in old tendon (15 with ≥2 peptides) (Table 3). STRING analysis revealed the GO cellular component “intermediate filament” was significantly increased in old tendon (Bonferroni adjusted *p* values 3.7E^−19^). Interestingly, in young tendon the SLRP family proteins fibromodulin, mimecan (osteoglycin), and asporin were significantly increased. By contrast, in old tendon, levels of several cellular proteins were increased, including several cytoskeletal keratins and gap junction proteins.

**TABLE 3:** **A number of differentially abundant proteins were identified by Progenesis^TM^ LC-MS software between young and old tendon. All proteins with a >2-fold change in normalized abundance are shown** A value of infinity is given when no peptides for a given protein in the group other than the highest mean condition were identified.

Highest mean condition	Accession	Description	Role	Peptide count	Max -fold change	ANOVA
						*p*
Young	XP_001503038.3	Protein disulfide-isomerase A3 isoform 1	ER enzyme-protein folding	2	Infinity	0.003
	XP_001491653.1	Asporin isoform 1	SLRP-collagen interaction	2	264.2	0.03
	XP_001916358.2	Neuroblast differentiation-associated protein AHNAK	Cell proliferation/differentiation	3	83.6	0.027
	XP_001497470.1	Histone H2B type 1-M isoform 1	DNA packaging protein	10	54.8	0.002
	XP_001500135.1	Histone H2B type 2-F	DNA packaging protein	10	54.5	0.006
	XP_001918010.1	Mimecan	SLRP-collagen interaction	4	29.4	0.002
	NP_001075246.1	Fibromodulin	SLRP-collagen interaction	14	21	0.008
	XP_001504528.1	Heat shock protein β-1	Antiapoptotic and anti-inflammatory	2	10.5	0.003
	XP_001915599.1	UPF0293 protein C16orf42	Unknown	1	Infinity	0.031
	XP_001493624.2	ras-related C3 botulinum toxin substrate 1	Cell growth, cytoskeletal organization	1	Infinity	0.004
	XP_001490052.2	Integrin β-2	Cell attachment	1	Infinity	0.003
	XP_001500627.1	Spectrin α chain, brain isoform 1	Cytoskeletal protein	1	Infinity	0.001
	XP_001496993.1	Peptidyl-prolyl cis-trans isomerase A isoform 1	Enzyme-protein folding	1	666.2	0.009
Old	XP_001504448.1	Keratin, type II cytoskeletal 75 isoform 1	Intermediate filament protein	11	Infinity	0.001
	XP_001497733.1	Keratin, type I cuticular Ha5	Intermediate filament protein	5	Infinity	0.001
	XP_001504468.1	Keratin, type II cytoskeletal 5 isoform 1	Intermediate filament protein	14	20	0.027
	XP_001504484.1	Keratin, type II cytoskeletal 73 isoform 1	Intermediate filament protein	8	16.6	0.047
	XP_001917840.1	Junction plakoglobin	Cell junction protein	2	9.9	0.049
	XP_001499854.1	Keratin, type I cytoskeletal 10	Intermediate filament protein	24	8.9	0.012
	XP_001496987.1	Keratin, type I cytoskeletal 14	Intermediate filament protein	10	8.9	0.006
	XP_001489373.1	Major allergen Equ c 1	Transporter protein	2	8.5	0.003
	XP_001504488.2	Keratin, type II cytoskeletal 2 epidermal	Intermediate filament protein	11	7.3	0.026
	XP_001494629.3	Keratin, type II cytoskeletal 1	Intermediate filament protein	12	6.3	0.032
	XP_001494427.2	Keratin, type II cuticular Hb5	Intermediate filament protein	12	4	0.029
	XP_001504460.2	keratin, type II cytoskeletal 6B	Intermediate filament protein	14	3.6	0.016
	XP_001916241.2	Desmoglein-1	Cell junction protein	2	2.1	0.012
	XP_001916548.2	Desmoplakin	Cell junction protein	1	Infinity	0
	NP_001093583.1	Secretory phospholipase A2	Lipid metabolism	1	7091.7	0.009
	NP_001075966.1	Major allergen Equ c 1 precursor	Transporter protein	1	779.5	0.001
	XP_001502526.1	arf-GAP with SH3 domain, ANK repeat and PH domain-containing protein 2	Vesicular transport	1	5.6	0.005

##### Western Blotting

**FIGURE 4.**
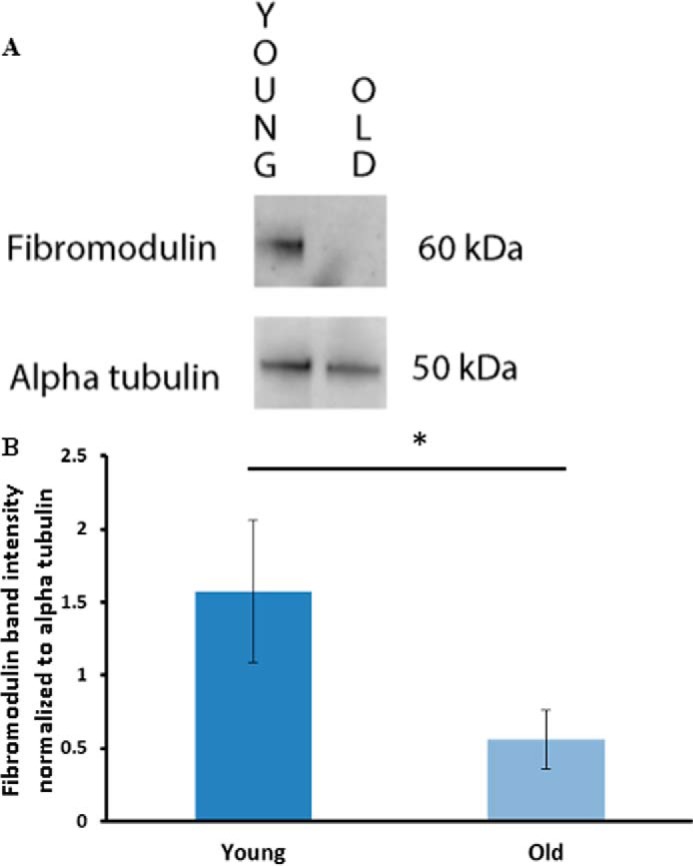
**Effect of age on fibromodulin levels in tendon fascicles.** *A*, representative blots of fibromodulin and α-tubulin in samples from young and old horses. *B*, Western blot analysis of fibromodulin normalized to α-tubulin demonstrated a significant reduction in fibromodulin with age. The histogram represents mean pixel intensity ±S.E., *n* = 3; *, *p* < 0.05.

Western blot analysis of normal young and old tendon fascicles confirmed the proteomic data by demonstrating a significant reduction in fibromodulin levels in older tendon (*p* < 0.04; Student's *t* test) (Fig. 4).

##### Differential Gene Expression

**FIGURE 5.**
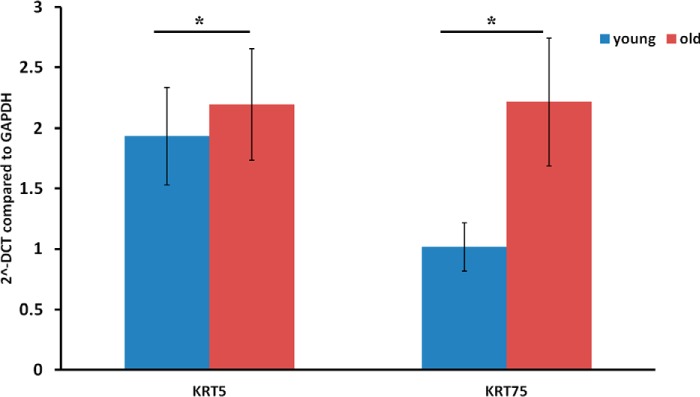
**Gene expression of KRT5 and KRT75 in normal young and old tendon.** Data are represented as 2 ΔCt (*2^-DCT*) compared with GAPDH. Histograms represent the means ± S.E. of mean. *, *p* < 0.05. Data were evaluated using Student's *t* test after log transformation for normalization (*n* = 7).

To investigate the increase in keratins in old normal tendon, RT-PCR was undertaken on an independent cohort of tendon from normal young and old donors using primer pairs for the genes KRT5 and KRT75. There was a significant increase in expression of both these genes in old normal tendon mirroring the protein expression changes (Fig. 5).

##### Injured Tendon

Soluble protein content corrected to fascicle wet weight was 34.5 ± 18.3 μg/ml in young injured tendon and 31.4 ± 12.4 in old injured tendon. SDS-PAGE analysis of guanidine-soluble proteins revealed a greater number of bands in injured tendon compared with normal (Fig. 1). A total of 278 proteins were identified in combined injured tendon samples; 250 had a significant Mascot score of >19. This was significantly greater than the number of proteins identified in normal tendon (*p* < 0.01). Supplemental Table 1 provides detailed information on the identification of peptides mapped to each protein and the corresponding Mascot scores. In diseased tendon 188 ± 2 and 150 ± 50 proteins were identified in young and old tendon, respectively. A large number of neopeptides were identified for proteoglycans and collagens in injured tendon, with many more neopeptides identified in young diseased than in old diseased samples (Table 2, supplemental Table 3). PCA at both the peptide (principal component of 36%) and protein levels revealed separation between young and old samples. However, the young samples were more tightly clustered than the old samples. There were 26 proteins at significantly higher levels in young injured tendon (23 with ≥2 peptide) (Table 4). DAVID identified the term acetylation as significantly increased in this protein set (supplemental Table 4). However, STRING did not find any protein-protein interaction within this set.

**TABLE 4:** **A number of differentially abundant proteins were identified by Progenesis^TM^ LC-MS software between diseased young and old tendon** All proteins with a >2-fold change in normalized abundance are shown. All proteins were at higher levels in the young diseased tendon.

Accession	Description	Role	Peptide count	Max fold change	ANOVA (*p*)
XP_001490861.2	Cofilin-2	Actin disassembly	2	631.4	0.003
XP_001493508.1	Tenascin-N	Facilitates neural growth*^a^*	4	18.2	0.019
XP_001501829.1	α-Crystallin B chain	Increase cell tolerance to stress*^a,b^*	8	12.7	0.004
XP_001503950.1	Tubulointerstitial nephritis antigen 1 isoform 1	Cysteine peptidase-ECM turnover	2	7.4	0.004
NP_001075421.1	α-Fetoprotein precursor	Plasma protein*^a^*	1	5.1	0.017
XP_001503085.1	Actin, aortic smooth muscle isoform 1	Cell motility	20	5.1	0.015
XP_001915023.1	Cysteine-rich protein 2	Inhibits inflammatory pain	1	5.0	0.009
XP_001917554.1	Procollagen C-endopeptidase enhancer 2	Enhances cleavage of pro-collagen*^a,b^*	3	4.4	0.003
XP_003363861.1	Heat shock 70-kDa protein 1	Protects cells from thermal stress	4	4.2	0.004
XP_001503538.2	Target of Nesh-SH3 isoform 4	ECM organisation	1	3.3	0.001
XP_001916170.2	protein SZT2	Involved in oxidative stress	5	3.2	0.020
XP_001915664.2	Microfibrillar-associated protein 5	Associates with elastic fibres, stabilizes procollagen	2	3.1	0.050
XP_001494523.1	Polymerase I and transcript release factor isoform	Transcription regulation	9	3.0	0.029
XP_001916600.1	Insulin growth factor binding protein-6	Carrier protein for IGF-1	3	3.0	0.033
NP_001229384.1	SH3 domain binding glutamic acid-rich protein like 3	Nuclear/cytoplasmic protein	3	2.9	0.004
XP_001503725.1	Annexin A6	Regulation of membrane traffic	4	2.8	0.025
XP_001498580.3	Integrin α-V	Cell-cell & cell-matrix interactions^a^	3	2.6	0.009
XP_001914753.2	Catalase	Prevents cells from oxidative damage	22	2.5	0.007
XP_001914918.2	Fibulin-2	Interacts with elastic fibres	13	2.3	0.030
XP_001504528.1	Heat shock protein β1	Protects cells from thermal stress	7	2.2	0.043
XP_001916279.2	ATP synthase subunit α, mitochondrial	ATP synthesis	4	2.1	0.032
XP_001496987.1	Keratin, type I cytoskeletal 14	Intermediate filament protein*^a^*	3	2.0	0.002

*^a^* Previously identified in developing tendon (25).

*^b^* Previously identified in healing tendon (24).

## DISCUSSION

We have performed a comprehensive proteomic analysis of healthy tendon tissue, identifying age-related alterations to the proteins present within the tendon matrix. The results support the hypothesis demonstrating distinct proteomic profiles in young and old tendon with decreased levels of several SLRPs and increases in intermediate filament proteins as a result of aging. In addition, a number of ECM protein fragments produced by fragmentation of the original peptide by enzymatic cleavage between two amino acids, which we have termed “neopeptides,” were identified in this study, and we propose these are related to specific cleavage sites. We have also assessed the proteomic profile of young and old injured tendon, demonstrating increased matrix fragmentation in disease and distinct proteomic profiles between age groups.

In both young and old normal samples, STRING analysis of the proteins present within the GndHCl-soluble extract revealed two connected clusters of proteins involved in collagen fibril and ECM organization as would be expected in tendon tissue. The most abundant collagen identified in the GndHCl-soluble extract was collagen type VI. Although few studies have investigated the role of collagen type VI in tendon, Izu *et al.* (40) showed that type VI collagen is localized to the pericellular region and is likely to play a role in collagen fibrillogenesis. Furthermore, it has been demonstrated that collagen VI mutant mice have abnormal collagen fibrils and exhibit muscle and tendon defects similar to those seen in human muscular dystrophy (41), suggesting that this minor collagen plays a crucial role in normal tendon function. A significant role for type VI collagen in tendon function is supported by these data.

Other proteins identified within the GndHCl-soluble extract include members of the thrombospondin family (COMP, thrombospondin-4 and -5) and several of the small SLRPs (decorin, fibromodulin, prolargin). Thrombospondins are known to regulate cell-matrix interactions, but their specific role in tendon has not been extensively studied. COMP is thought to catalyze collagen fibrillogenesis and stabilize the collagen network (42), and COMP levels have been correlated with tendon mechanical properties (43).

Decorin is the most abundant and the most studied of the SLRPs within tendon. However, its role is yet to be fully established. Both decorin and fibromodulin are involved in fibrillogenesis (1), and decorin may also play a role in transfer of force between collagen fibrils (44), although this function is contentious (45). To the authors' knowledge this is the first work to identify the presence of prolargin within tendon tissue. This class II SLRP is able to bind to type I collagen and is postulated to anchor basement membranes to the connective tissue (46).

Although there was no overall decrease in the concentration of soluble proteins extracted from normal tendon with increasing age as determined by the Bradford assay, silver staining of bands on one-dimensional gels of soluble protein extracts appeared to decrease with aging. Assessment of the normalization factor used by Progenesis during relative quantification revealed higher normalization factors for old samples even though a fixed amount of protein (based on Bradford assay results) was loaded. This suggests that the Bradford assay may have provided an incorrect estimation of sample protein content, as reported previously (47), but the reasons for this are unclear. Taken together, these results suggest that in aged tendon protein extractability was reduced, suggesting that the matrix in aged samples is more resistant to degradation, with more proteins remaining trapped within the insoluble portion of the matrix. There were also age-related differences in the pepsin-released portion of the samples, with more collagenous polypeptide bands evident in young samples (Fig. 2). These findings are supported by previous work which has shown that with aging partially degraded collagen accumulates within the matrix of the SDFT, which may be due to increased levels of glycation, rendering the matrix more resistant to degradation (14).

There were no alterations in the levels of the major matrix components with increasing age. This supports our previous studies which have shown that tendon water, collagen, and glycosaminoglycan content of the equine SDFT do not change with aging (14). However, there was a reduction in levels of several less-abundant proteins with increasing age in normal tendon, including several SLRPS (fibromodulin, mimecan, asporin). The age-related reduction in fibromodulin was further confirmed by Western blotting. These proteoglycans interact with collagen and have all been shown to regulate collagen fibrillogenesis and fibril diameter (1, 48–50). Heat shock protein β1, also known as heat shock protein 27, also decreased with aging. Heat shock proteins have anti-apoptotic and anti-inflammatory roles and have also been shown to increase in tendinopathy, where it is suggested they may play a role in tendon healing (51). The reduction in levels of these proteins with aging may, therefore, affect maintenance and repair of matrix in old tendon and could contribute to the increased risk of tendon injury in aged individuals.

There were also alterations in levels of several cellular proteins with aging, with decreases in histones and integrins and increases in keratins and gap junction proteins with aging. Previous studies have shown that cellularity within the SDFT does not change with aging (12), suggesting that these alterations may reflect an age-related change in cell phenotype. The majority of proteins that increased with age were keratins and gap junction proteins. Although some cuticular keratins were identified, possibly due to contamination from hair during dissection, the majority of keratins identified were cytoskeletal, and their increase was confirmed at the gene expression level. STRING analysis revealed these proteins belong to the GO cellular component intermediate filament. These filaments form cytoskeletal networks that are important in maintenance and regulation of cell tension, providing support for the plasma membrane during contact with cells and extracellular matrix (52). The increases in intermediate filament proteins observed with aging may indicate an increase in cell stiffness, which could result in an altered cell response to tensile loading in aged tendons. Although the specific roles of cytoskeletal keratins in tendon are not well understood, it has been previously demonstrated that keratin 1 and 10 are localized to the basement membrane epithelium recently identified around tendon (53). This basement membrane is thought to regulate cell migration and maintain tendon functional integrity (53). A change in the levels of cytoskeletal components suggests differences in their mechanical behavior with aging.

In addition, a number of previously documented as well as novel neopeptides were identified in this study. It is likely that those found in both young and old normal samples could be due to normal ECM turnover. However, they may represent proteolytic cleavage occurring as a consequence of subclinical pathological degradation, or they could be neopeptides produced during tissue processing as cell death can release intracellular proteases. Protease activity during processing seems unlikely as attempts to mitigate this were made through the chilling and rapid post-mortem dissection of the limbs and snap-freezing of tissues.

It could be hypothesized that the neopeptides identified in normal young tendon alone represent ECM fragments produced by normal tissue remodeling, which is altered with aging and injury. We identified the COMP neopeptide NTVMECDACGMQP↓A in young tendon only. This cleavage pattern has been attributed to the activity of a disintegrin with thrombospondin motifs (ADAMTS)-5 in mouse.^5^ Conversely, although a greater number of neopeptides were identified in young than in old tendon, neopeptides present only in old tendon may represent important events in tendon aging and provide an insight into the underlying mechanisms that consequently increase the risk of tendon injury in aged individuals. Interestingly the COMP neopeptides F↓CFSQENIIWANLR and C↓PDGTPSPCHEK, identified in old healthy tendon only, have been recently identified within equine SDFT tissue (18). The neopeptide F↓CFSQENIIWANLR was evident in subacute SDFT injury and also after IL-1β stimulation of macroscopically normal equine SDFT explants. One proposed theory of aging is the “senescent secretory phenotype” (54). Accumulation of specific cells, which secrete increased amounts of cytokines, contributes to cell aging. The identification of this neopeptide in both studies could indicate that there may be enhanced production of cytokines in tendon aging. This could provide a direct link between aging and inflammation similar to that proposed in cartilage (55).

In the study by Dakin *et al.*, C↓PDGTPSPCHEK was evident in both normal SDFT and in control explants maintained in culture for 24 h (11). Due to the wide age range used in their study, it would be interesting to undertake further work to assess if this neopeptide represents a specific aging biomarker and if so, at which age it appears.

A number of collagen neopeptides were also identified. Interestingly we previously proposed that an inability to remove partially degraded collagens from the tendon matrix may lead to reduced mechanical competence in aging tendon (14). The collagen I, VI, and XII neopeptides identified here in only the old normal tissue could support this hypothesis. In young tendon a number of collagen VI and XII fragments were also evident. This could be explained by the role of collagen VI in fibrillogenesis (40) and collagen XII in ECM organization (56) and represent normal matrix turnover due to weaker ECM interactions of these collagens in young tendon. Indeed, in developing chick tendon it has been demonstrated that collagen VI is predominantly located to the interfascicular matrix (56). This is interesting as our previous work has shown that the interfascicular matrix plays an important role in SDFT function but becomes stiffer in aged tendon (30, 57), which may be due to a reduced ability to turn over this matrix with aging.

It was not possible to directly compare the proteome of normal and injured tendon, as these samples were analyzed at different times. However, there are some clear differences between normal and injured states. Although the soluble protein content was lower, a greater number of proteins were identified in injured tendon compared with normal. This may be because soluble protein content was normalized to fascicle wet weight; it has previously been shown that tendinopathic tissue has a higher water content than normal tendon (58), which would result in a relatively lower protein content when normalizing to wet weight. Furthermore, additional bands were visible in samples from injured tendon, which are likely to be due to increased matrix degradation and fragmentation (Fig. 1). An increase in cellularity may also contribute to the increase in the number of proteins identified in injured tendon. Many of the proteins identified in diseased tendon were cellular, and it is well established that cell numbers are increased in injured tendon (59–61). Furthermore, a larger number of neopeptides were identified in injured tendon, indicating a greater degradation of collagens and proteoglycans with disease.

It is also apparent that the proteomic profile differs with age in injured tendon. A number of proteins were detected at higher levels in young compared with old diseased tendon. These include several cellular proteins, which have roles in protection of cells from stress and synthesis and stabilization of matrix proteins (see Table 5). Furthermore, several of these proteins have been identified in developing tendon (25) and in artificially induced tendon lesions (24). The higher levels of these proteins in young diseased tendon may, therefore, represent a healing response, which appears to be limited in old diseased tendon (63). A larger number of neopeptides was also identified in young injured tendon, suggesting a greater ability to degrade damaged regions of the matrix. This may further explain why aged tendons are more at risk of injury, as a failed healing response is likely to lead to the accumulation of microdamage and subsequent injury. However, it is unclear if this failed healing response is due to a decreased ability of tendon cells to synthesize and degrade damaged regions of the matrix in aged tendons or whether the matrix is more resistant to degradation due to age-related glycation.

There are several limitations to this study that need to be considered. The high levels of collagenous proteins in tendon mean that it is difficult to detect proteins present at a low abundance. Future studies could use hexapeptide peptide library protein normalization (62), which would allow identification of low abundance proteins. Alternatively, absolute protein quantification using QconCat technology could be used in an artificially aged *in vitro* model (20), which would allow the analysis of a greater number of samples. In addition, proteins present may not have been identified as they could not be extracted from the matrix, which is highly resistant to degradation. Furthermore, it is evident that protein extractability is altered with aging in normal tendon; therefore, some of the age-related alterations identified could be because the proteins were not extracted from the matrix. However, if this were the case it would be expected there would be a global decrease in protein levels with aging, which was not observed. It should also be considered that some of the proteins identified, particularly keratinous proteins, may be due to contamination from skin and hair. Care was taken during dissection to ensure minimal contamination, and as the majority of keratins identified were cytoskeletal rather than cutaneous, this is unlikely to be a major source of contamination. Furthermore, we have confirmed the increased keratin levels with aging at the mRNA level.

## CONCLUSIONS

Although proteomic analysis is fast becoming a standard technique to study many soft tissues, few studies have attempted to use this technique to characterize tendon tissue. In the current study we have demonstrated age-related alterations in several proteins within normal tendon, with decreases in proteins that play a role in ECM organization and increases in cytoskeletal proteins. We have further demonstrated an altered proteomic profile in injured tendon, with significantly more proteins identified and a greater degree of matrix fragmentation. We have also shown a decrease in levels of proteins associated with reduction of cell stress and increased matrix synthesis with aging in injured tendon. This study has increased the knowledge of molecular events associated with tendon degradation characteristic of aging and injury and identified peptides that may be useful as biomarkers of tendon injury. These findings suggest that maintenance and repair of tendon tissue may be reduced in aged individuals, resulting in an impaired healing response, and may help to explain why the risk of injury increases with aging.

## Supplementary Material

Supplemental Data

## Footnotes

5 P. Holden, personal communication.

## Footnotes

4 The abbreviations used are:

ECM: extracellular matrix
ANOVA: analysis of variance
COMP: cartilage oligomeric matrix protein
DAVID: Database for Annotation, Visualization, and Integrated Discovery
GdnHCl: guanidine hydrochloride
GO: gene ontology
SDFT: superficial digital flexor tendon
SLRP: small leucine-rich proteoglycan
STRING: Search Tool for Retrieval of Interacting Genes/Proteins
Bis-Tris: 2-[bis(2-hydroxyethyl)amino]-2-(hydroxymethyl)propane-1,3-diol.

## References

[1] Yoon J. H. Halper J. (2005) Tendon proteoglycans: biochemistry and function. J. Musculoskelet. Neuronal Interact. 5, 22–34 15788868

[2] Riley G. (2008) Tendinopathy: from basic science to treatment. Nat. Clin. Pract. Rheumatol. 4, 82–89 18235537 10.1038/ncprheum0700

[3] Grant T. M. Thompson M. S. Urban J. Yu J. (2013) Elastic fibres are broadly distributed in tendon and highly localized around tenocytes. J. Anat. 222, 573–579 23587025 10.1111/joa.12048 PMC3666236

[4] Thorpe C. T. Birch H. L. Clegg P. D. Screen H. R. (2013) The role of the non-collagenous matrix in tendon function. Int. J. Exp. Pathol. 94, 248–259 23718692 10.1111/iep.12027 PMC3721456

[5] Kastelic J. Galeski A. Baer E. (1978) The multicomposite structure of tendon. Connect Tissue Res. 6, 11–23 149646 10.3109/03008207809152283

[6] Kujala U. M. Sarna S. Kaprio J. (2005) Cumulative incidence of Achilles tendon rupture and tendinopathy in male former elite athletes. Clin. J. Sport Med. 15, 133–135 15867554 10.1097/01.jsm.0000165347.55638.23

[7] Clayton R. A. Court-Brown C. M. (2008) The epidemiology of musculoskeletal tendinous and ligamentous injuries. Injury 39, 1338–1344 19036362 10.1016/j.injury.2008.06.021

[8] Birch H. L. Bailey A. J. Goodship A. E. (1998) Macroscopic “degeneration” of equine superficial digital flexor tendon is accompanied by a change in extracellular matrix composition. Equine Vet J. 30, 534–539 9844973 10.1111/j.2042-3306.1998.tb04530.x

[9] Tully L. J. Murphy A. M. Smith R. K. Hulin-Curtis S. L. Verheyen K. L. Price J. S. (2014) Polymorphisms in TNC and COL5A1 genes are associated with risk of superficial digital flexor tendinopathy in National Hunt Thoroughbred racehorses. Equine Vet J. 46, 289–293 23906005 10.1111/evj.12134

[10] September A. V. Cook J. Handley C. J. van der Merwe L. Schwellnus M. P. Collins M. (2009) Variants within the COL5A1 gene are associated with Achilles tendinopathy in two populations. Br. J. Sports Med. 43, 357–365 18443036 10.1136/bjsm.2008.048793

[11] Dakin S. G. Dudhia J. Smith R. K. (2014) Resolving an inflammatory concept: the importance of inflammation and resolution in tendinopathy. Vet. Immunol. Immunopathol. 158, 121–127 24556326 10.1016/j.vetimm.2014.01.007 PMC3991845

[12] Birch H. L. Bailey J. V. Bailey A. J. Goodship A. E. (1999) Age-related changes to the molecular and cellular components of equine flexor tendons. Equine Vet. J. 31, 391–396 10505954 10.1111/j.2042-3306.1999.tb03838.x

[13] Avery N. C. Bailey A. J. (2005) Enzymic and non-enzymic cross-linking mechanisms in relation to turnover of collagen: relevance to aging and exercise. Scand. J. Med. Sci. Sports 15, 231–240 15998340 10.1111/j.1600-0838.2005.00464.x

[14] Thorpe C. T. Streeter I. Pinchbeck G. L. Goodship A. E. Clegg P. D. Birch H. L. (2010) Aspartic acid racemization and collagen degradation markers reveal an accumulation of damage in tendon collagen that is enhanced with aging. J. Biol. Chem. 285, 15674–15681 20308077 10.1074/jbc.M109.077503 PMC2871433

[15] Riley G. P. Harrall R. L. Constant C. R. Chard M. D. Cawston T. E. Hazleman B. L. (1994) Glycosaminoglycans of human rotator cuff tendons: changes with age and in chronic rotator cuff tendinitis. Ann. Rheum. Dis. 53, 367–376 8037495 10.1136/ard.53.6.367 PMC1005351

[16] Smith R. K. Zunino L. Webbon P. M. Heinegård D. (1997) The distribution of cartilage oligomeric matrix protein (COMP) in tendon and its variation with tendon site, age, and load. Matrix Biol. 16, 255–271 9501326 10.1016/s0945-053x(97)90014-7

[17] Rousseau J. C. Delmas P. D. (2007) Biological markers in osteoarthritis. Nat. Clin. Pract. Rheumatol. 3, 346–356 17538566 10.1038/ncprheum0508

[18] Dakin S. G. Smith R. K. Heinegård D. Önnerfjord P. Khabut A. Dudhia J. (2014) Proteomic analysis of tendon extracellular matrix reveals disease stage-specific fragmentation and differential cleavage of COMP. J. Biol. Chem. 289, 4919–4927 24398684 10.1074/jbc.M113.511972 PMC3931053

[19] Wilson R. Diseberg A. F. Gordon L. Zivkovic S. Tatarczuch L. Mackie E. J. Gorman J. J. Bateman J. F. (2010) Comprehensive profiling of cartilage extracellular matrix formation and maturation using sequential extraction and label-free quantitative proteomics. Mol. Cell. Proteomics 9, 1296–1313 20190199 10.1074/mcp.M000014-MCP201 PMC2877988

[20] Peffers M. J. Beynon R. J. Clegg P. D. (2013) Absolute quantification of the human osteoarthritic secretome. Int. J. Mol. Sci. 14, 20658–20681 24132152 10.3390/ijms141020658 PMC3821636

[21] Jiang Y. Liu H. Li H. Wang F. Cheng K. Zhou G. Zhang W. Ye M. Cao Y. Liu W. Zou H. (2011) A proteomic analysis of engineered tendon formation under dynamic mechanical loading in vitro. Biomaterials 32, 4085–4095 21402406 10.1016/j.biomaterials.2011.02.033

[22] Han G.-Y. Park S.-A. Kim J.-H. Lee E.-K. Kim H.-J. Seo Y.-K. Park J.-K. Kim C.-W. (2011) Effects of vibration on the proteome expression of anterior cruciate ligament cells. Exp. Biol. Med. 236, 783–789 10.1258/ebm.2011.010358 21693654

[23] Jielile J. Jialili A. Sabirhazi G. Shawutali N. Redati D. Chen J. Tang B. Bai J. Aldyarhan K. (2011) Proteomic analysis of differential protein expression of Achilles tendon in a rabbit model by two-dimensional polyacrylamide gel electrophoresis at 21 days postoperation. Appl. Biochem. Biotechnol. 165, 1092–1106 21800109 10.1007/s12010-011-9327-7

[24] Jielile J. Aibai M. Sabirhazi G. Shawutali N. Tangkejie W. Badelhan A. Nuerduola Y. Satewalede T. Buranbai D. Hunapia B. Jialihasi A. Bai J. P. Kizaibek M. (2012) Active Achilles tendon kinesitherapy accelerates Achilles tendon repair by promoting neurite regeneration. Neural Regen. Res. 7, 2801–2810 10.3969/j.issn.1673-5374.2012.35.008 PMC4190862 25317130

[25] Smith S. M. Thomas C. E. Birk D. E. (2012) Pericellular proteins of the developing mouse tendon: a proteomic analysis. Connect Tissue Res. 53, 2–13 21851252 10.3109/03008207.2011.602766 PMC3771084

[26] Kasashima Y. Takahashi T. Smith R. K. Goodship A. E. Kuwano A. Ueno T. Hirano S. (2004) Prevalence of superficial digital flexor tendonitis and suspensory desmitis in Japanese thoroughbred flat racehorses in 1999. Equine Vet. J. 36, 346–350 15163043 10.2746/0425164044890580

[27] Innes J. F. Clegg P. (2010) Comparative rheumatology: what can be learnt from naturally occurring musculoskeletal disorders in domestic animals? Rheumatology 49, 1030–1039 20176567 10.1093/rheumatology/kep465

[28] Lui P. P. Maffulli N. Rolf C. Smith R. K. (2011) What are the validated animal models for tendinopathy? Scand J. Med. Sci. Sports 21, 3–17 20673247 10.1111/j.1600-0838.2010.01164.x

[29] Ely E. R. Avella C. S. Price J. S. Smith R. K. Wood J. L. Verheyen K. L. (2009) Descriptive epidemiology of fracture, tendon, and suspensory ligament injuries in National Hunt racehorses in training. Equine Vet. J. 41, 372–378 19562899 10.2746/042516409x371224

[30] Thorpe C. T. Udeze C. P. Birch H. L. Clegg P. D. Screen H. R. (2013) Capacity for sliding between tendon fascicles decreases with ageing in injury prone equine tendons: a possible mechanism for age-related tendinopathy? Eur. Cell Mater. 25, 48–60 23300032 10.22203/ecm.v025a04

[31] Lichtwark G. A. Wilson A. M. (2005) *In vivo* mechanical properties of the human Achilles tendon during one-legged hopping. J. Exp. Biol. 208, 4715–4725 16326953 10.1242/jeb.01950

[32] Biewener A. A. (1998) Muscle-tendon stresses and elastic energy storage during locomotion in the horse. Comp Biochem. Physiol B. Biochem. Mol. Biol. 120, 73–87 9787779 10.1016/s0305-0491(98)00024-8

[33] Legerlotz K. Riley G. P. Screen H. R. (2010) Specimen dimensions influence the measurement of material properties in tendon fascicles. J. Biomech. 43, 2274–2280 20483410 10.1016/j.jbiomech.2010.04.040 PMC2935962

[34] McLean L. Hurst J. L. Gaskell C. J. Lewis J. C. Beynon R. J. (2007) Characterization of cauxin in the urine of domestic and big cats. J. Chem. Ecol. 33, 1997–2009 17924168 10.1007/s10886-007-9354-6

[35] Franceschini A. Szklarczyk D. Frankild S. Kuhn M. Simonovic M. Roth A. Lin J. Minguez P. Bork P. von Mering C. Jensen L. J. (2013) STRING v9.1: protein-protein interaction networks, with increased coverage and integration. Nucleic Acids Res. 41, D808–D815 23203871 10.1093/nar/gks1094 PMC3531103

[36] Peffers M. Liu X. Clegg P. (2013) Transcriptomic signatures in cartilage ageing. Arthritis Res. Ther. 15, R98 23971731 10.1186/ar4278 PMC3978620

[37] Birch H. L. Worboys S. Eissa S. Jackson B. Strassburg S. Clegg P. D. (2008) Matrix metabolism rate differs in functionally distinct tendons. Matrix Biol. 27, 182–189 18032005 10.1016/j.matbio.2007.10.004

[38] Dakin S. G. Werling D. Hibbert A. Abayasekara D. R. Young N. J. Smith R. K. Dudhia J. (2012) Macrophage subpopulations and the lipoxin A4 receptor implicate active inflammation during equine tendon repair. PLoS ONE 7, e32333 22384219 10.1371/journal.pone.0032333 PMC3284560

[39] Webbon P. M. (1977) A post mortem study of equine digital flexor tendons. Equine Vet. J. 9, 61–67 862604 10.1111/j.2042-3306.1977.tb03981.x

[40] Izu Y. Ansorge H. L. Zhang G. Soslowsky L. J. Bonaldo P. Chu M.-L. Birk D. E. (2011) Dysfunctional tendon collagen fibrillogenesis in collagen VI null mice. Matrix Biol. 30, 53–61 20951202 10.1016/j.matbio.2010.10.001 PMC3778658

[41] Pan T.-C. Zhang R.-Z. Markova D. Arita M. Zhang Y. Bogdanovich S. Khurana T. S. Bönnemann C. G. Birk D. E. Chu M.-L. (2013) COL6A3 protein deficiency in mice leads to muscle and tendon defects similar to human collagen VI congenital muscular dystrophy. J. Biol. Chem. 288, 14320–14331 23564457 10.1074/jbc.M112.433078 PMC3656288

[42] Halász K. Kassner A. Mörgelin M. Heinegård D. (2007) COMP acts as a catalyst in collagen fibrillogenesis. J. Biol. Chem. 282, 31166–31173 17716974 10.1074/jbc.M705735200

[43] Smith R. K. Gerard M. Dowling B. Dart A. J. Birch H. L. Goodship A. E. (2002) Correlation of cartilage oligomeric matrix protein (COMP) levels in equine tendon with mechanical properties: a proposed role for COMP in determining function-specific mechanical characteristics of locomotor tendons. Equine Vet. J. Suppl. 34, 241–244 12405694 10.1111/j.2042-3306.2002.tb05426.x

[44] Redaelli A. Vesentini S. Soncini M. Vena P. Mantero S. Montevecchi F. M. (2003) Possible role of decorin glycosaminoglycans in fibril to fibril force transfer in relative mature tendons: a computational study from molecular to microstructural level. J. Biomech. 36, 1555–1569 14499303 10.1016/s0021-9290(03)00133-7

[45] Provenzano P. P. Vanderby R. (2006) Collagen fibril morphology and organization: implications for force transmission in ligament and tendon. Matrix Biol. 25, 71–84 16271455 10.1016/j.matbio.2005.09.005

[46] Bengtsson E. Mörgelin M. Sasaki T. Timpl R. Heinegård D. Aspberg A. (2002) The leucine-rich repeat protein prelp binds perlecan and collagens and may function as a basement membrane anchor. J. Biol. Chem. 277, 15061–15068 11847210 10.1074/jbc.M108285200

[47] Weist S. Eravci M. Broedel O. Fuxius S. Eravci S. Baumgartner A. (2008) Results and reliability of protein quantification for two-dimensional gel electrophoresis strongly depend on the type of protein sample and the method employed. Proteomics 8, 3389–3396 18646011 10.1002/pmic.200800236

[48] Kalamajski S. Aspberg A. Lindblom K. Heinegård D. Oldberg A. (2009) Asporin competes with decorin for collagen binding, binds calcium, and promotes osteoblast collagen mineralization. Biochem. J. 423, 53–59 19589127 10.1042/BJ20090542

[49] Kalamajski S. Oldberg A. (2009) Homologous sequence in lumican and fibromodulin leucine-rich repeat 5–7 competes for collagen binding. J. Biol. Chem. 284, 534–539 19008226 10.1074/jbc.M805721200

[50] Tasheva E. S. Koester A. Paulsen A. Q. Garrett A. S. Boyle D. L. Davidson H. J. Song M. Fox N. Conrad G. W. (2002) Mimecan/osteoglycin-deficient mice have collagen fibril abnormalities. Mol. Vis. 8, 407–415 12432342

[51] Millar N. L. Murrell G. A. (2012) Heat shock proteins in tendinopathy: novel molecular regulators. Mediators Inflamm. 2012, 436203 23258952 10.1155/2012/436203 PMC3507314

[52] Lodish H. B. A Zipursky SL (2000) Molecular Cell Biology, 4th Ed., pp. 806–813, W. H. Freeman, New York

[53] Taylor S. H. Al-Youha S. Van Agtmael T. Lu Y. Wong J. McGrouther D. A. Kadler K. E. (2011) Tendon is covered by a basement membrane epithelium that is required for cell retention and the prevention of adhesion formation. PLoS ONE 6, e16337 21298098 10.1371/journal.pone.0016337 PMC3027644

[54] Campisi J. (2005) Senescent cells, tumor suppression, and organismal aging: good citizens, bad neighbors. Cell 120, 513–522 15734683 10.1016/j.cell.2005.02.003

[55] Loeser R. F. (2010) Age-related changes in the musculoskeletal system and the development of osteoarthritis. Clin. Geriatr. Med. 26, 371–386 20699160 10.1016/j.cger.2010.03.002 PMC2920876

[56] Zhang G. Young B. B. Birk D. E. (2003) Differential expression of type XII collagen in developing chicken metatarsal tendons. J. Anat. 202, 411–420 12739618 10.1046/j.1469-7580.2003.00174.x PMC1571097

[57] Thorpe C. T. Udeze C. P. Birch H. L. Clegg P. D. Screen H. R. (2012) Specialization of tendon mechanical properties results from interfascicular differences. J. R. Soc. Interface 9, 3108–3117 22764132 10.1098/rsif.2012.0362 PMC3479922

[58] de Mos M. van El B. DeGroot J. Jahr H. van Schie H. T. van Arkel E. R. Tol H. Heijboer R. van Osch G. J. Verhaar J. A. (2007) Achilles tendinosis: changes in biochemical composition and collagen turnover rate. Am. J. Sports Med. 35, 1549–1556 17478653 10.1177/0363546507301885

[59] Södersten F. Hultenby K. Heinegård D. Johnston C. Ekman S. (2013) Immunolocalization of collagens (I and III) and cartilage oligomeric matrix protein (COMP) in the normal and injured equine superficial digital flexor tendon. Connect Tissue Res. 54, 62–69 23020676 10.3109/03008207.2012.734879 PMC3545546

[60] Kobayashi A. Sugisaka M. Takehana K. Yamaguchi M. Eerdunchaolu Iwasa E. K. Abe M. (1999) Morphological and histochemical analysis of a case of superficial digital flexor tendon injury in the horse. J. Comp. Pathol. 120, 403–414 10208736 10.1053/jcpa.1998.0288

[61] Patterson-Kane J. C. Firth E. C. (2009) The pathobiology of exercise-induced superficial digital flexor tendon injury in thoroughbred racehorses. Vet. J. 181, 79–89 18406184 10.1016/j.tvjl.2008.02.009

[62] Dwivedi R. C. Krokhin O. V. Cortens J. P. Wilkins J. A. (2010)) Assessment of the reproducibility of random hexapeptide peptide library-based protein normalization. J. Proteome Res. 9, 1144–1149 20020779 10.1021/pr900608z

[63] Dakin S. G. Dudhia J. Werling N. J. Werling D. Abayasekara D. R. Smith R. K. (2012) Inflamm-Aging and Arachadonic Acid Metabolite Differences with Stage of Tendon Disease. PLoS ONE 7, e48978 23155437 10.1371/journal.pone.0048978 PMC3498370

