# Supplementary material for: Proteomic Analysis Reveals Age-related Changes in Tendon Matrix Composition, with Age- and Injury-specific Matrix Fragmentation
Source: J Biol Chem. 2014 Jul 30;289(37):25867–78. doi: 10.1074/jbc.M114.566554 (PMC4162187; doi:10.1074/jbc.M114.566554)
Supplement: Supplemental Data [file supp_289_37_25867__index.html]

Proteomic analysis reveals age-related changes in tendon matrix composition, with age-and 1 injury-specific matrix fragmentation — Proteomic Analysis Reveals Age-related Changes in Tendon Matrix Composition, with Age- and Injury-specific Matrix Fragmentation — Proteomics Reveals Changes with Aging and Injury in Tendons — Supplemental Data 

# Proteomic Analysis Reveals Age-related Changes in Tendon Matrix Composition, with Age- and Injury-specific Matrix Fragmentation

## Supplemental Data

**Files in this Data Supplement:**

- Supplementary Table 1 (.xlsx, 1.9 MB) - Detailed information of the identification of peptides mapped to each protein and corresponding Mascot scores
- Supplementary Table 2 (.xlsx, 206 KB) - DAVID &#x26; GO - normal tendon
- Supplementary Table 3 (.xlsx, 24 KB) - Details of neopeptides identified in young and old, normal and injured tendon
- Supplementary Table 4 (.xlsx, 312 KB) - DAVID &#x26; GO - injured tendon
